# “The one that got away”- therapists’ experiences when patients suddenly drop out from psychotherapy: a thematic analysis

**DOI:** 10.1186/s40359-026-03958-z

**Published:** 2026-01-15

**Authors:** Niclas Kullgard, Melinda Börjesson, Johanna Carlsson, Rolf Holmqvist, Gerhard Andersson

**Affiliations:** 1 Department of Behavioural Sciences and Learning, Linköping University, Linköping, Sweden; 2 Department of Biomedical and Clinical Sciences, Linköping University, Linköping, Sweden; 3 Department of Clinical Neuroscience, Karolinska Institutet, Stockholm, Sweden

**Keywords:** Psychotherapy, Sudden dropout, Thematic analysis, Therapist experiences

## Abstract

**Objective:**

The objective of the study was to examine how therapists in clinical practice experience sudden dropouts from psychotherapy.

**Method:**

We interviewed 12 licensed psychotherapists regarding sudden dropouts. The interviews were analysed using thematic analysis (TA).

**Results:**

Three main themes and seven subthemes were identified. The first main theme was: *Struggling to understand and explain the dropout.* The subthemes were: *“the patients’ problem was too difficult”*,* “trouble cooperating” and “problematic emotional bond”.* The second main theme was: *Continuing when something is wrong.* The subthemes were: “*difficulties maintaining presence”*,* “emotional withdrawal and sense of failure as a therapist”.* The last third main theme was: *Therapists processing feelings after the dropout.* The subthemes were: “*doubting your own capacity”*,* “to be abandoned”*,* “sense of relief and lessons learned”.*

**Conclusions:**

Sudden treatment dropouts often elicit negative emotions such as guilt, shame, and sorrow. They may also bring a sense of relief. Abrupt termination of therapy can affect therapists both professionally and personally, with the impact sometimes persisting long after the client has dropped out from therapy.

**Supplementary Information:**

The online version contains supplementary material available at 10.1186/s40359-026-03958-z.

## Clinical or methodological significance of this article

Dropout seems to affect therapists mainly by leading to guilt, a sense of inadequacy or relief. The observations may be important as topics for supervision and in educational settings during psychotherapy training.

## Introduction

Premature termination of psychotherapy is common in clinical practice and has been recognized in the clinical research literature. In a previous survey we explored therapist-rated prevalence of premature dropout, the reasons behind and the experienced consequences following a premature dropout. Therapists reported experiencing self-doubt and powerlessness following treatment discontinuation [1]. The emotional impact on therapists when patients end therapy prematurely has not been much investigated in previous research. There is also a need for a more comprehensive understanding of the mechanisms underlying premature endings, particularly to prevent them when they may be harmful for the patient. In the present study, we explored psychotherapists’ experiences when therapy is terminated abruptly and without a clear explanation from the patient.

There is no established consensus-based definition of dropout from psychotherapy. Hatchett and Park [2] described four distinct methods to classify and measure dropout: therapist judgement, termination by failure to attend the last scheduled session, not attending a predetermined number of sessions, and failure to return after an intake appointment. These differences have led to difficulties when evaluating to what degree patients drop out from psychotherapy. One example is when a patient does not show up for the next session in the beginning of therapy before setting goals and does not continue with the therapy. This type of dropout can be interpreted as a dropout, but another interpretation is that the therapy has not even started and that it should not be regarded as a dropout. In addition to the definitional inconsistencies used to classify premature termination — and the resulting challenges in comparing studies employing different criteria — questions remain about how to understand the processes leading to early termination and what occurs in therapies when patients discontinue. Another issue, as noted by Norcross and Lambert [3, 4], is that comparisons of psychotherapy outcomes, such as rates of premature termination, across specific treatment methods or therapy durations often overlook the influence of therapist factors and the therapeutic relationship. This omission is problematic as that these factors appear to be associated with treatment outcomes [3, 4]. When compared with agreed upon termination of therapy, a sudden dropout is a special and sometimes difficult form of dropout. The abrupt ending of therapy, without any explanation, can be hard to grasp and emotionally challenging for therapists, as they may not understand what happened or the reasons behind. Dropout may occur when the therapy does not give expected results. It can lead to suffering for patients, therapists and health organizations [5]. Data from outcome studies in psychotherapy suggest that approximately one of five therapies ends due to dropout [6, 7]. In our previous study, therapists working in regular clinical practice estimated that 8.9% of their clients had dropped out without notice [1]. These results suggest that there may be differences in frequencies of dropout between clinical practice settings and research studies.

In all forms of psychotherapy ending therapy is part of the therapeutic process. Thus, ending therapy can be very different depending on the type of therapy (for example open-ended or time-limited), and how the termination is handled. It may be more or less explicit, for example as a part of the therapeutic contract or when goals have been reached. Sometimes the ending occurs without further notice and when the therapy is far from completed. One aspect of dropout is how the therapeutic contract is set up, for example if there is a statement regarding duration of therapy and/or treament goals (e.g., reduced symptoms). Added to that there may be limits of therapy duration based on costs and insurance coverage.

Several patient-related factors have been identified as being associated with dropout in psychotherapy outcome studies, including low socioeconomic status [8], substance abuse, criminal involvement, severe psychiatric disorders [8, 9], low educational attainment and a weak therapeutic alliance [5, 6, 10–15]. In addition, persons with low socioeconomic status report less control and influence over difficult life situations that are likely to lead to psychological stress, which then increases the risk of dropping out [11, 14, 16]. From a psychodynamic process perspective, Bergmann [17] described two overarching patterns emerging from clinical observations of dropout. One pattern concerned termination due to death or medical circumstances, while the other reflected experiences of hostility toward the therapy and/or the therapist. Particularly noteworthy in this latter pattern were that patients expressed feelings of dissatisfaction and disappointment, which manifested as overt hostility toward both the therapeutic process and the therapist [17]. Dropout may also be understood as a defensive process against increasing emotional closeness and dependency, and/or as an enactment of power within the therapeutic relationship in relation to the therapist. From this perspective, dropout can function as a means of avoiding affective experiences associated with termination, allowing the patient to assume control over both the therapeutic process and its ending. For instance, patients may experience emotions that they perceive as difficult or impossible to articulate within the therapeutic dialogue, and which are instead expressed through the act of dropping out. [17].

Previous meta-analyses and psychotherapy outcome studies shows that a strong therapeutic alliance is related to better outcome, and that a weak alliance is related to dropout [18–20]. A meta-analysis by Sharf & Primavera regarding alliance and psychotherapy outcome showed that a weak alliance was correlated with dropout [15]. Less is known about how and in what way the therapeutic relationship influences dropout. Therefore, qualitative research has been recommended as a way to increase understanding of the processes behind dropouts [5, 21]. One important aspect concern how the quality of the therapeutic alliance influences the patient’s decision to drop out, as well as the significance of identifying and addressing alliance ruptures. In our previous study therapists rated the alliance as good in most therapies even when patients prematurely drop out from therapy [1]. This indicates that strains in the alliance are difficult to detect, especially when a patient withdraws from therapy that is ongoing. Although a sudden dropout from therapy seems hasty, there may be a longer withdrawal process when the patient considers ending therapy without telling the therapist.

The negative impact on the therapist, both as professional and as a person has been investigated with fairly consistent findings. Nissen-Lie et al. [22] noted that the emotional burden on psychotherapists can influence therapy outcome. Farber [23] reported that dropout was the third greatest source of stress for therapists, including psychologists, psychiatrists, and social workers. Guy [24] described therapists feeling hurt, abandoned, betrayed and/or disappointed following dropout. Ogrodniczuk, Joyce, and Piper [25] pointed out increased lack of confidence and diminished sense of self-worth. Pekarik [26] described therapists feeling dissatisfied, lonely, isolated, and burned out when patients drop out. A dropout may however also sometimes lead to a sense of relief, especially if the therapy has been difficult and with poor therapeutic alliance. At the same time it may for the same therapist also lead to self-doubt and guilt [27, 28]. In our previous study [1] therapists identified self-doubt and powerlessness as the most common feelings following a premature dropout. To cope with negative feelings’ therapists may overcompensate through distancing themselves from emotional engagement or become overly attached and motivated to work with their patients [24]. In one study therapists were asked to provide an answer for why a hypothetical patient had dropped out from therapy. The results showed that therapists tended to blame the patient for the dropout [29]. This may be interpreted as a coping strategy when therapists distance themselves from their own responsibility for the dropout in order to protect their self-esteem. In a more recent study these findings were not replicated, indicating that blaming the patient could occur in some cases, but that the issue is more complex [30]. In another study therapists were asked to reflect upon the impact on themselves professionally as well as personally. The study showed that problems in the therapy were related to the therapeutic alliance, difficulties to understand strains in the alliance as markers for dropout, and a lack of professional impact [31]. Overall, these reports clearly suggest that dropout is an emotional challenge for therapists with the exception being when a dropout leads to a sense of relief and potentially less workload.

Several limitations remain in the existing literature, particularly regarding the processes leading up to dropout. There is also a limited understanding of which aspects of the therapeutic alliance may be especially salient in this process, as well as how psychotherapists and patients experience and manage their emotional responses both during the period preceding dropout and in its aftermath. The aim of this study was to explore how psychotherapists working in routine clinical practice experience and respond to patient dropout. We had no restrictions on what type of patients or therapy orientation was given, only that the therapy had to be initiated and agreed upon (e.g., not supportive counselling, assessment only or one session consultations). Our definition of a sudden dropout was: *When a patient stops coming without notice to an ongoing psychotherapy.*

## Method

The study used qualitative methodology [32]. Open-ended interviews were conducted and the approach was mainly inductive but acknowledged the assumption that dropout would largely be a negative experience. In the interviews the respondents were asked to describe and reflect upon their own experiences of specific dropouts based on their own clinical context and experiences. The analysis was inspired by a realist approach although latent interpretation of codes and themes was possible when it seemed to make content more meaningful. The coders had received psychotherapy training and worked with psychotherapy using a relational psychodynamic approach. Our own pre-existing knowledge including theoretical background was difficult to ignore when making meaning of codes and sequences from interviews and creating themes. To decrease the risk that the themes were too much influenced by our professional and theoretical background we strived to use semantic interpretation of codes and themes.

### Participants

As the purpose was to investigate psychotherapists experiences of patient dropping out, we narrowed the sample in terms of being a licensed psychotherapist and practicing psychotherapy working in Sweden [32]. This was done since there are licensed therapists who do not practice and may not have done so for many years following training. We interviewed 8 female and 4 male licensed psychotherapists who had experienced client dropout from psychotherapy. They had practiced psychotherapy between 1 and 22 years (M = 8.3 yrs, Mdn = 5.5 yrs), and the proportion of psychotherapy practice ranged between 30 and 100% of their current work. The occupational background included eight social workers, two psychologists, one occupational therapist and one nurse. Five participants worked in primary care, four in psychiatry, two in private practice and one in an online primary care setting. Regarding the psychotherapy orientation eight stated Cognitive Behavioural Therapy (CBT) and seven mainly Psychodynamic Psychotherapy (PDT), with some therapists working with both CBT and PDT.

We expected 12 respondents to be sufficient for the aims of the study [33]. The therapists were primarily recruited via announcements in Swedish professional social media groups on Facebook in which the psychotherapists were active. We also contacted primary care and psychiatric clinics nearby and sent out information about the study via email. Interested psychotherapists were asked to contact us via email. More information was sent to the therapists about the study and if they still were interested, a written formal consent was obtained, and the interview was scheduled.

### Procedure for data collection

All interviews were conducted online via Zoom. This was done because of the ongoing Covid-19 restrictions at the time of the interviews. All recorded interviews were subsequently transcribed. Settings for Zoom were end-to-end encryption to ensure that no third part could access the interviews. The interviews lasted between 30 and 96 min (on average 53 min), and the total time of the recordings was 637 min.

### Interview protocol

Based on previous literature and clinical practice an interview protocol was developed and tested in a pilot interview. The interview protocol has not been used in any other study. After the pilot interview the protocol was slightly modified. It contained questions on background factors like profession, workplace, years as practitioner, gender and main therapeutic orientation used. Questions regarding experiences of dropping out were mostly open-ended and with possibilities for the interviewer to pose follow-up questions on topics that had been talked about during the interview. The aim of the questions was to create a dialogue during which the informant could easily discuss the topic and not be restricted by shame, guilt or a sense of failure as therapist. During the interviews, the therapists reflected on their experiences of a patient that had dropped out from an ongoing therapy. They were willing to share their feelings and that the topic was engaging. During the interviews they could change their minds and/or add additional thoughts and reflections.

### Analyses

The researchers were all licensed psychologists and/or psychotherapists who work clinically with psychotherapy. Four of were trained in relational psychodynamic psychotherapy (PDT) and one in cognitive behavioural therapy (CBT). All had experienced dropout in various degree and were interested in understanding the phenomena of sudden dropout. The material was analysed by using thematic analysis (TA)[34]. The purpose of using TA as method for analysis was to find patterns and to describe experiences that were not only explicitly communicated in the interviews. Braun and Clarke described six phases in the analysis of data: *familiarisation with your data*; *generate initial codes*; *searching for themes*; *reviewing themes*; *defining and naming themes* and *produce the report*.

In the first phase, *familiarisation with data*, the interviews were transcribed and read, which gave an initial understanding of the content. *Coding* was done by three of the authors (MB, JC and NK). In the coding process, citations from interviews were the most common code. In some cases, latent codes were created based on what was said by the interviewee and the coders the emotional understanding of the context in the sequence that was coded. In the third phase, *searching for themes*, codes were organised in preliminary themes which were discussed to reach consensus of themes. There were initially 24 themes. In the process of finding the essence of a specific theme there was an intent to actively move from what was said explicitly by the responders to a deeper understanding made by the coder. The process can be described as moving back and forth between the explicit data and our own personal and theoretical understanding of the codes. The fourth phase is “*reviewing themes”*. In this phase we tried to find themes that stood out and “told” us something that illustrated therapists’ experiences of terminations when patients drop out. Also, we tried to single out more distinct themes when themes overlapped. During this phase, themes were presented to an experienced group of six psychotherapists who were also researchers in clinical psychology. The purpose of sharing themes was to investigate the correspondence and coherence of themes and subthemes [35]. The results of their input were that themes were corresponding well but in some cases were renamed to increase correspondence. After reviewing the data, three themes were selected based on what was most noteworthy. In the fifth phase, *”refining*,* defining and naming”*, we clarified the meaning of the themes. When there were doubts about a theme, we either divided the theme into additional themes or put the theme “*on hold*” until we were more certain about what the theme described. Sometimes a theme became a bit clearer when we let it go for a while and then came back to the theme. The sixth phase is *“writing up”.* The process concerned selecting themes for the paper and to end the process of finding new angles or themes. A theme was selected for writing up when it judged to provide a new perspective and if it added to the research questions asked.

## Results

### Thematic analysis

**Fig. 1:**
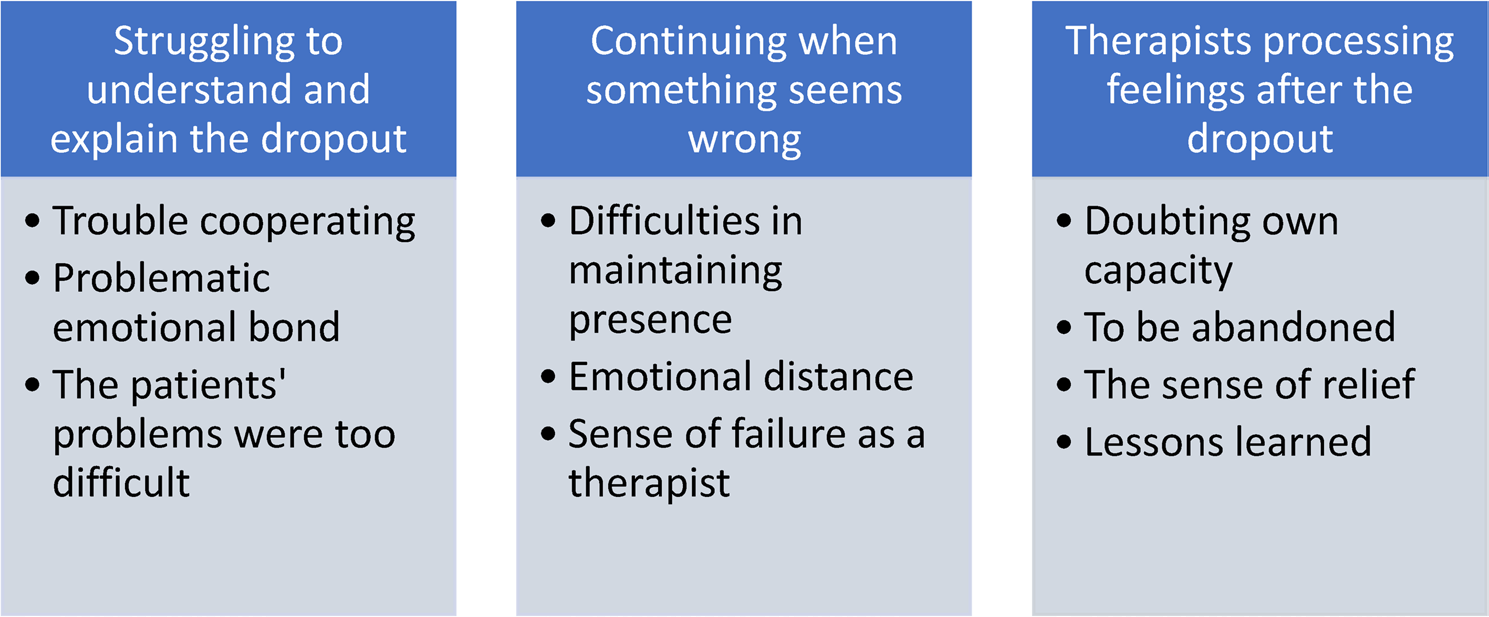
Themes and subthemes regarding therapists’ experiences of dropout

The thematic analysis resulted in three main themes, with three subthemes for each theme (Fig. 1), reflecting different ways of understanding and handling sudden dropouts. The themes were: *Struggling to understand and explain*,* Continuing when something seems wrong*,* and Therapists processing feelings after the dropout.*

Together the themes reflect a temporal sequence. The first sequence was focused on attempts to understand what had caused the dropout, the second on the emotional strains during the therapy, and finally the third theme covering the aftermath with effects on the therapist as a person as well as a professional. Each theme and subtheme will be described and elaborated on below.

### Struggling to understand and explain the dropout

This theme describes how therapists’ understanding and reasoning about **why** the dropout had occurred. In the three subthemes dropout was explained by factors primarily related to patients such as being too difficult, psychiatric diagnoses, personality and/or motivation. Some therapists described that they ruminate about different hypothesis regarding the patient’s motives for the dropout The rumination contained considerable self-blame, therapeutic competence and self-degeneration.

#### Trouble cooperating

Some therapists were concerned about the ability to cooperate with the patient due to differences *between* therapists and patients. The perceived difference was viewed as an additional explanation for the dropout.

Therapists also described subtle problems and did not primarily experience any explicit ones but experienced that something was not working well. One therapist described this process in the following quote.

I work with a person [who dropped out] and have a pretty good alliance or something like that, then suddenly I feel that something happened and even if I asked the patient, I would not get an answer regarding what it was that was not working well. (Therapist 11)

The quotation appears to illustrate a transition from a phase of collaborative engagement and mutual positive affect to a moment in which a subtle disruption in the emotional bond emerges, prompting the therapist to sense that something is amiss, despite the absence of any explicit discussion about it.

#### Problematic emotional bond

Some therapists were concerned about the emotional bond and that this was an additional explanation for the dropout. The concern was sometimes mentioned in association with the therapeutic alliance, or interpersonal chemistry.

To put it simple there may be so that the chemistry was not there, or there was no alliance. (Therapist 4)

This quotation captures the process of working together with an evolving suspicion that there is something wrong in the cooperation and also maybe a gist of sadness for being left by the patient.

#### The patients were too difficult for therapy

Therapists described how they tried to understand the dropout and elaborated on reasons that related to the severity of patients’ personality problems, difficult psychological experiences and/or psychiatric diagnosis. One aspect of the dropout was that it was mostly due to the patients’ difficulties.

There seemed to be almost no reference to the therapist themselves, or that there was a strain in the therapeutic relationship. A narrative regarding dropout was that reasons for dropout due to some “objective” fact for example the patients’ emotional availability or psychiatric diagnosis. Sometimes it was as if the patient made a rational choice to drop out and not do the work required in therapy. One therapist described this in the following citation.

Patients who dropout or want to change therapist often have a troubled personality and want something else and don’t want to work with themselves. (Therapist 7)

Another aspect of the patient being too difficult was more straightforward and generalized. For example, there were specific psychological experiences, for example trauma or personality disorder, that could explain the dropout. One example is this quotation from one therapist.

It is patients that have a severe disturbance, not that they have been psychotic or something like that, but a personality disorder that have chosen not to come back. (Therapist 11)

Some therapists described that patients had acted as if they had misunderstood the nature of therapy and therefore dropped out. It was described as if patients wanted more of an everyday conversation and friendly support or therapy that could change everything in the patient’s life. The therapists seemed more inclined to “work” with patients’ difficulties. It seems like there were different agendas for the sessions.

Patients feel that “this is not helping”, or maybe they have too high expectations or demands regarding what therapy can achieve. (Therapist 5)

The patients’ emotional availability was another narrative for the dropout that the therapists repeatedly mentioned. When the patient was emotionally unavailable the possibility of working in therapy seemed to be especially challenging or even impossible. One therapist described the person as follows.

It was a person who for several years had been very disconnected emotionally, not perceiving that it was possible to connect to any emotions at all, either positive or negative. (Therapist 6)

The subtheme again suggested that therapists explained the dropout from their own perspective, that there are patients who because of their psychological limitations as emotional availability, psychological trauma or specific diagnosis drop out from therapy. This one-sided explanation seemed sometimes to reduce guilt or own responsibility for the therapist, so that it was possible to continue working without too much emotional pressure and a preserved positive professional self-image.

### Continuing when something seems wrong

In the second theme the therapists described emotional challenges *during* the therapy. The descriptions dealt with both explicit and emotional struggles when working with a patient who seemed to withdraw or not respond to therapy. In the interviews implicit feelings of sadness, for example not being able to help, were conveyed. A citation by one therapist describes the core of this theme:

I was working with something and then it suddenly feels like there is a distance and I don’t really understand, the patient won’t tell you and then the patient doesn’t come back. (Therapist 11)

This quote indicates that the therapist keeps working while the emotional distance evolved and then being left.

#### Therapists have difficulties maintaining presence

Difficulties were described as something that occurred inside the therapists due to the interaction in therapy. The therapists described getting preoccupied with some aspects of the therapy and less present in the moment. This could evolve from an interest to understand *why* something did not work out and then losing presence.

Of course, there is a great deal of self-examination to grasp what happened. Was I too busy? If it is supposed to be ratings or if it is homework, and then you follow up and check how has this *worked out?* Then maybe I became too preoccupied in those parts of therapy and missed the presence needed and didn’t meet up the emotional need. (Therapist 7)

Sometimes it was described as losing focus on a specific therapeutic task that was agreed upon. In the narratives there seemed to be a tendency to withdraw from tasks or goals and maybe a shift for the therapist into continuing therapy without asking the patient for information about reasons for not doing what was agreed upon.

#### Emotional distance

In this subtheme therapists described that they became *emotionally* distant in the therapy. The distance was described as something evolving during therapy and that something was created, which was detected in the sessions. In this quotation a therapist described this process.

I don’t know if there was something I did, but there was certainly something that happened between us that created some kind of distance. From a good contact that became more emotionally distant. Anyway, I didn’t realize us becoming more emotionally distant, and it was already too late cause I didn’t notice anything when it happened. (Therapist 11)

Another description of distance was a sense of not reaching a personal bond because of lack of personal and maybe emotional closeness. The difficulties to establish closeness seemed to be due to a feeling of not getting along. This description of closeness appeared to be related to a feeling of differences in personality. This was described in the following quotation.

The patient feels that he or she is not getting along with me as a person. (Therapist 5)

In this theme narratives regarding distance in therapy was described as an evolving process from good to bad and as more distinct described differences in personality.

#### Sense of failure

When working with a patient who later dropped out therapists described a sense of failure as a professional therapist. Sometimes also as a person. These two aspects were entangled so that the professional and personal effects sometimes was hard to separate. Another interpretation could be that the loss of control over the therapy process led the therapist to take the blame for the dropout. The loss of control and the sense that the therapy was not proceeding well seems to elicit guilt and shame. One therapist described the sense of failure in the following quotation:

Then there is a discomfort, like what happened? What did I do, I was about to say wrong, but I mean it really feels like you missed something, that it was a misjudgement in some way. (Therapist 7)

The sense of failure could also be about yourself as a therapist, for example your methods and/or general skill. One therapist described it like this:

If you really think about it, reflect upon it, then you think, damn now you really messed up. I try to find another point of view other than I failed in my skilfulness. (Therapist 1)

Sometimes the sense of failure was described as feelings of insecurity, both as a person or as a professional. The sense of failure seemed to be personal.

I don’t feel super skilled, which can give a feeling that your work is simply not that good. Maybe I question myself and get insecure concerning if I did something crazy or something like that. (Therapist 2)

The personalization of failure that was described seemed to leave some therapists feeling failed as a person rather than something related to methodological issues that failed. This seemed to add to a professional and personal stress leaving some therapists doubting of their profession.

### Therapists processing feelings after the dropout

In the third theme therapist’s describe challenges after the dropout. We formulated four subthemes based on the narratives: *Doubting own capacity*,* To be abandoned*,* Sense of relief*,* and Lessons learned*.

#### Doubting your capacity

In the first subtheme the therapists described doubts about their capacity as a therapist and sometimes as a person. They described self-blame for example not knowing better. Reasons for the dropout were ascribed to themselves as persons, and their specific competence regarding the patients’ problems and/or diagnosis. The self-blame was sometimes straightforward but also doubting reasons for the dropout and what went wrong. In this quotation the different kinds of doubt are described by one therapist.

Sometimes I doubt my capacity and overrate how important I am for the therapy, or I think “this person dropped out because of *me*”. Was it something that I didn’t do so well or was I really bad? These are thoughts that I easily get but I don’t really know why someone drops out, it could be any reason (Therapist 5)

The descriptions regarding professional competence or capacity seemed devaluing and left the therapist in doubt both as a person and as a professional. One therapist described the interface between the personal and professional self in the following quotation.

After the dropout I spent a lot of time, really long time, wondering about what went wrong, was it something that I did wrong. For me it just felt like a big failure. (Therapist 10)

In the quotation, the therapist also describes difficulties in letting go when the dropout was experienced as a significant failure, suggesting that it affected the personal sphere.

#### To be abandoned

In this second subtheme the therapists described experiences after being abandoned by the patient. The dropout was described as sudden and sometimes as a rejection. This quote highlights one of the therapists’ thoughts and emotions after the dropout being like a loss:

It was really, really hard, it was almost like, and I don’t want to exaggerate, but almost like someone died because it all happened very fast. (Therapist 10)

Another description was that therapists offered something helpful which the patient first accepted but then without a clear explanation discarded. One therapist described experiencing the dropout as a rejection.

Of course, it is hard. I offered something that someone discards and shows “thanks but no thanks”. (Therapist 1)

The sense that wonders about a dropout stayed within therapists after the therapy had ended was described. In this quotation this experience was described from one of the therapists.

When I work as a therapist with my patients I invest my time, emotions and ideas about how I can help this person in the best way. So, it [the dropout] affects me, I wonder what happened? This wonder becomes like a dedication. I become dedicated to what happened, was it something I said, could I have done something different. (Therapist 12)

#### The sense of relief

In this subtheme the therapist expressed a sense of relief after the dropout. Two aspects of relief were described: personal relief and getting help to end therapy. Personal relief seemed to reflect that therapists experienced not getting along with certain patients more personally. When the patient left therapy it was described as relieving that the therapist did not have to work with the patient. The following quotation describes one kind of relief after the dropout.

Sometimes you need to see people that you don’t get along with or that become triggering for me. In some ways it feels *good* that I don’t have to see that person anymore. (Therapist 4)

The second description of relief reflected that the therapist received “help” to end therapy from the patient and that they in that sense were relieved. It was as if the therapist wanted to end the therapy but did not know how. When the patient dropped out it was described as relief rather than something problematic. In this quotation one therapist describes this experience of getting help to end therapy.

If I’m straightforward, it was helpful for ending therapy, that the patient decided to quit. (Therapist 2)

The quotation appears to highlight how the therapist, with the patient’s assistance, was able to end a therapeutic process that had not been experienced as beneficial and which the therapist had struggled to conclude independently.

#### Lessons learned

In this final subtheme the therapists described lessons learned from the dropout. They seemed sad and/or puzzled about being left behind without understanding what happened. The uncertainty seems to leave the therapist in doubt of what they did wrong. This quote describes both the wish to learn from the therapy and to know what happened:

I think of them [patients that dropout] more than others, how it worked out for them. I think “the one that got away”. I really would have wanted to know, even if the person doesn’t want to see me, what would I have needed to know or learn. (Therapist 1)

This theme and quote also reflect therapy as an ongoing learning process, not only for the patient but also for the therapist. When a sudden dropout occurs, the therapists were confused about what could be learned from the patient and therapy. The emotional experience of being left by the patient seems to get in the foreground for summing up what went on in therapy and how to learn from the ended therapy.

## Discussion

Three main themes were identified in this study when investigating psychotherapists’ experiences when patients drop out from an ongoing psychotherapy: *Struggling to understand and explain the dropout*,* Continuing when something seems wrong*,* and therapists processing feelings after the dropout*. Although each story from the therapists was unique these themes recurred in several interviews. Overall, the therapists did not refer to some of the factors commonly identified in psychotherapy outcome research, such as low socioeconomic status [8] or lower educational attainment [9]. Other factors were more salient in the therapists’ narratives, as their accounts and interpretations of dropout foregrounded severe psychological difficulties [8] and challenges within the therapeutic alliance, particularly those involving a problematic emotional bond [6, 10–15].

The two first themes may seem contradictory. That the therapists accused themselves as well as their patients for the dropout. Our understanding is that this reflects different aspects of dropout, and that there is no single explanation explaining dropout. The first theme reflected how therapists try to understand what had happened in therapy that preceded the dropout and why this had happened. Different types of understanding were described, and an observation was that therapists find explanations for the dropout related to their patients. For example, that the patient was particularly difficult or problematic in their relatedness or emotional availability. Our findings suggest that therapists try hard to grasp the difficulties on their own. There were no descriptions from the therapists trying to communicate their thoughts about the difficulties with the patient. Previous research shows that clinical judgement and decisions sometimes are shaped by heuristics and biases. This seems to be more common when therapists are faced with pressure and ambiguity [36]. Our findings also showed that the therapists described that when they feel insecure or under pressure, they relied on their own judgement but considered the possibility that more information could be obtained by exploring clients’ experiences during therapy. Indeed, there is emerging evidence that therapy can benefit from asking for feedback from the patient at every session including both the therapy process and how symptoms develop [37]. In the therapists’ description of what happened in therapy they used typical psychological language, for example that the patient had a specific diagnosis or emotional difficulties expressed in psychological terms. Maybe this is what most therapists do when they are challenged or a consequence of the interview setting as they knew that the interviewer also was a psychotherapist. It could be problematic if therapists too rigidly rely on pathology explanations, for example psychiatric diagnoses, if it leads to fewer questions when they suspect a dropout. This may create a problematic distance in the therapeutic alliance especially when it is not communicated. In a previous study on dropout therapists showed withdrawal rupture by not being curious and having trouble taking the patients perspective [38]. The emotional distance could be interpreted as a therapist withdrawal rupture, but this would have to be more thoroughly investigated. Another interpretation would be that the therapists *co-create* an emotional distance from the patient by trying to solve a shared problem themselves. It could be that therapists, when under pressure, use their theoretical knowledge to cope with heightened emotional distress such as self-blame, fear of rejection or lack of confidence. If the coping strategy then becomes more of a defence mechanism, helping the therapist survive the therapeutic interplay when under pressure, there is a risk that the therapist distance themselves from the patient. Therapists seemed to get entangled in the complexity of trying to understand and work with a patient who gradually withdraws from therapy.

In the second theme therapists more explicitly described working with patients when something seemed wrong. One aspect of the theme was the struggle to maintain presence when working with a patient who seems to withdraw from therapy. Some therapists described that an emotional distance could evolve without explicit cues indicating that there was something wrong or that the patient was in some ways dissatisfied. In these processes the therapists described a feeling of being a failure, rejected and that something was missing without being able to pinpoint the reasons behind. When emotionally challenged with this type of therapeutic interaction therapists described more excessive self-examination with focus on what they had said or done that created emotional distance. As in the first theme therapists described that it was their task to discover possible reasons for the distance. In this process some therapists could become less focused on the goals of therapy [19]. One interpretation is that the therapist must find ways to stay interested in the therapy while feeling shame and wanting to end or escape from the therapy. In order to retain the professional self, the therapist may need some type of psychological protection. This could be described as a defence mechanism, particularly in situations where the therapist experiences a sense of ineffectiveness or fails to establish contact with the patient, leading to feelings of rejection.

The third theme focused on the aftermath from a dropout. The therapists described dropouts was something that the reflect upon and wonder what happened even long after the dropout. This corresponds with the findings in the study by Piselli et al. [31]. Therapists in our study also doubted their capacity as a psychotherapist, including self-blame, difficulties in maintaining hope and sadness after the dropout, but sometimes also a sense of relief. This was in line with our previous quantitative findings where therapists reported self-doubt after premature dropout [1]. Also, in a study by Nissen-Lie et al. [22] self-doubt was reported as negative if the therapists doubted themselves as a person. Abrupt and surprising dropouts seemed to lead to more difficult feelings for the therapists. Since the therapist is left alone to understand the dropout it is hard to get the facts. When faced with a separation the therapists may blame themselves and devalue their competence. These feelings resemble other separations when one is being abandoned with no possibility to ask the one who left what happened. The negative feelings that therapists described such as self-blaming, self-doubt and hopelessness are common in times of crisis, especially sudden deaths such as accidents or suicide [39]. “Forbidden” feelings like relief seem more related to separations due to difficult circumstances, like prolonged sickness or leaving harmful relationships. In life in general we often talk to significant others to solve our dilemmas or get help to understand and process difficult feelings. But for therapists there may be limited room for this and close colleagues or supervision is the only option for privacy reasons. Some therapists in private practice may not have close colleagues. Further a reluctance to talk about such issues may also play a role as dropout seems to be related to a sense of failure, shame and guilt.

Finally, the findings of this study underscore the centrality of the emotional bond in therapists’ work with patients experienced as difficult. The results further suggest that this bond does not simply dissolve when therapy is terminated abruptly; rather, it appears to persist within the therapist in the form of self-doubt, sorrow, worry, shame, and guilt. An additional finding is that therapists tended to experience these affective responses in isolation, seldom sharing them with patients, supervisors, or colleagues. The study highlights that such feelings are commonly experienced among therapists and may contribute to deeper understanding when articulated and shared, whereas remaining isolated may be more problematic.

### Strengths and limitations

In this study we interviewed clinical psychotherapists without any restrictions regarding patient psychiatric diagnoses, psychotherapy method, or length of the psychotherapies. The patients who dropped out described in the interviews were their own patients in clinical practice. This could be considered as strengthening the ecological validity. The interviews were characterized by depth, which was observed when more difficult feelings or thoughts about the dropout evolved during the course of the interviews.

Some limitations should be considered. The focus of the interview was on the dropout and there were no explicit questions about the patients or the therapeutic process leading to the dropout. A second limitation is the one-sided focus on the therapists’ experiences of dropout, with insufficient knowledge about the patients’ thoughts and feelings regarding dropout. There is a need to study the perspective of patients to more fully understand how and why a dropout occurs. A final limitation is that we did not consider the organizational perspective, for example if there are certain demands such as case load or clinical priorities on the therapists that may increase the likelihood of a dropout.

### Future research

The negative effects related to dropout for patients, therapists, and health organisations makes it important to understand processes in therapy that precede dropout. This study indicates that dropout has negative effects on the therapist, such as emotional burden and distress. One implication may be that more difficult patients are not selected for therapy due to the professional and emotional risk for the therapist in case they drop out. This could lead to the risk that patients regarded as a potential non-responders/dropouts from therapy are dismissed. Further research is needed to understand why patients decide to rapidly end treatment but also how the emotional burden on therapist is handled. Another area for further research is how the alliance evolves in therapies which ends in a dropout. Research has mainly focused on patients in therapies that are completed. Our study indicates that there may be withdrawal ruptures that are not repaired, and it may even be that this is initiated by the therapist when they *feel* that the patient does not want to be in therapy.

## Conclusions

In conclusion, sudden dropouts seem to impact therapists both professionally and personally long after a dropout has occurred. This should be considered in the training of psychotherapists as it is seldom covered in spite of being common. Sudden dropouts tend to evoke feelings of guilt, shame, and sorrow, but they may also bring a sense of relief. The inability to work through those feelings with the patient can make it difficult to understand what is behind the decision to drop out. Without a collaborative process, it becomes challenging to fully understand the underlying reasons behind the dropout and apply those insights to decrease the risk of sudden dropouts.

## Supplementary Information

Supplementary Material 1.

## Data Availability

The data that support the findings of this study are available from Linköping University, but restrictions apply to the availability of these data, which were used under ethical approvement for the current study and so are not publicly available. The data are, however, available from the authors upon reasonable request.
