# Supplementary material for: “The one that got away”- therapists’ experiences when patients suddenly drop out from psychotherapy: a thematic analysis
Source: BMC Psychol. 2026 Jan 15;14:120. doi: 10.1186/s40359-026-03958-z (PMC12849187; doi:10.1186/s40359-026-03958-z)
Supplement: Supplementary file 1 — Supplementary Material 1. [file 40359_2026_3958_MOESM1_ESM.docx]

**Guide for Interview.**

**Short presentation of the interviewer and the frames for the interview.**

1. The interview will take about an hour and will be recorded, only with sound.
2. Short definition of what kind of drop out we are interested in. *The dropouts that we are investigating are when an agreement has been done for a psychotherapy and that the patient suddenly either stops showing up or says that he/she don’t want to continue and the therapy. But are also* *interested if you have any other type of dropout that you have experience of.*

**Background**

1. For how many years have you been practising psychotherapy?
2. To what extend do you practise psychotherapy within your current employment?
3. In what kind of organisation/s do you work with psychotherapy?
4. What is your professional background?
5. Which methods of psychotherapy do you work with?
6. Can you tell me about your experiences of patients dropping out of therapy?
7. Have you suspected that a patient is about to drop out of therapy?
8. Do your thoughts about drop out during therapy affect your way of working in therapy?
9. What do you think was reasons for patients dropping out of therapy?
10. How do you usually react when a patient dropped out of therapy?
11. How has dropouts affected your professional role as a therapist?
12. How do you talk about patients dropping out of therapy on your workplace?
13. Do you have any concluding thoughts on drop out that we have not talked about and that you whish to share?

● Vad är det första du kommer att tänka på när det kommer till terapiavhopp?

● Hur ser dina erfarenheter av avhopp ut?

● Har du misstänkt att avhopp kan komma att ske innan det eventuellt hänt? Hur då?

● Påverkar dina tankar kring avhopp ditt sätt att arbeta på?

● Vad tror du att avhoppen har berott på?

● Hur brukar du reagera på avhoppen?

● Hur har avhoppen påverkat dig i din yrkesroll?

● Hur pratas det om avhopp på din arbetsplats?

● Nu har vi berört de områden som vi är intresserade av att undersöka men jag tänkte

höra med dig om det är något du känner att vi missat att fråga om, eller om det är

något som du själv har tänkt på kopplat till avhopp som vi inte har berör
